# BCL2 Inhibitor (ABT-737): A Restorer of Prednisolone Sensitivity in Early T-Cell Precursor-Acute Lymphoblastic Leukemia with High *MEF2C* Expression?

**DOI:** 10.1371/journal.pone.0132926

**Published:** 2015-07-14

**Authors:** Sachiko Kawashima-Goto, Toshihiko Imamura, Chihiro Tomoyasu, Mio Yano, Hideki Yoshida, Atsushi Fujiki, Shinichi Tamura, Shinya Osone, Hiroyuki Ishida, Akira Morimoto, Hiroshi Kuroda, Hajime Hosoi

**Affiliations:** 1 Department of Pediatrics, Kyoto Prefectural University of Medicine, Graduate School of Medical Science, Kyoto, Japan; 2 Department of Pediatrics, Matsushita Memorial Hospital, Moriguchi, Japan; 3 Department of Pediatrics, Kyoto City Hospital, Kyoto, Japan; 4 Department of Pediatrics, Jichi Medical University School of Medicine, Shimotuke, Japan; European Institute of Oncology, ITALY

## Abstract

Early T-cell precursor-acute lymphoblastic leukemia (ETP-ALL) has been identified as a high-risk subtype of pediatric T-cell acute lymphoblastic leukemia (T-ALL). Conventional chemotherapy is not fully effective for this subtype of leukemia; therefore, potential therapeutic targets need to be explored. Analysis of the gene expression patterns of the transcription factors in pediatric T-ALL revealed that *MEF2C* and *FLT3* were expressed at higher levels in ETP-ALL than typical T-ALL. Using human T-ALL and BaF3 cell lines with high expression levels of *MEF2C*, the present study tested whether the BCL2 inhibitor (ABT-737) restores the sensitivity to prednisolone (PSL), because MEF2C causes PSL resistance, possibly by augmenting the anti-apoptotic activity of BCL2. Treatment with PSL and ABT-737 caused a significant reduction in the IC50 of PSL in the *MEF2C*-expressing LOUCY cells, in addition to the *MEF2C*-transduced BaF3 cells, but not in the non-*MEF2C*-expressing Jurkat cells. The combination treatment significantly accelerated the killing of primary leukemic blast cells of ETP-ALL with high expression levels of *MEF2C*, which were co-cultured with murine stromal cells. These findings suggest that BCL2 inhibitors may be a therapeutic candidate *in vivo* for patients with ETP-ALL with high expression levels of *MEF2C*.

## Introduction

T-cell acute lymphoblastic leukemia (T-ALL) is an aggressive leukemia, accounting for 10–15% of childhood ALL cases. With a wide use of intensive chemotherapy, the prognosis of childhood T-ALL has improved; nearly 80% of patients can currently be cured [1–3]. However, approximately one-fifth of children with T-ALL succumb to the disease. To improve the prognosis of these patients, it is important to clarify any unrecognized poor risk-related biological characteristics that may contribute to drug resistance.

To date, studies have identified a distinct biological subtype of T-ALL, i.e., early T-cell precursor ALL (ETP-ALL), which is characterized by a gene expression profile recapitulating that of normal early T-cell precursors (ETPs) [4]. ETPs arise from multipotent bone marrow progenitors, which possess T, B, and myeloid potentials. Selectively potent ETPs arise from T and myeloid potentials, but not B potentials, which represent the earliest thymic progenitors [5].

ETP-ALL is characterized by the lack of expression of CD1a and CD8, weak or absent expression of CD5, and aberrant expression of myeloid and hematopoietic stem cell markers, such as CD13, CD33, CD34 and CD117 [4]. In these cases, conventional chemotherapy was not fully effective for ETP-ALL. Patients with ETP-ALL show high remission failure or hematological relapse, according to studies at the St Jude Children’s Research Hospital and the Associazione Italiana Ematologia Oncologia Pesiatrica (AIEOP) [4], indicating that novel therapeutic measures should be explored. The gene expression pattern of the transcription factors related to the differentiation of lymphoid and myeloid cells in ETP-ALL was assessed in an attempt to identify potential therapeutic targets for this particular subtype of pediatric ALL. The present findings suggest that a BCL2 inhibitor could reverse the drug resistance and increase the sensitivity to prednisolone (PSL) in the treatment of EPT-ALL.

## Materials and Methods

### Ethical Statement

The written informed consent was obtained from the guardians of patients, according to the Declaration of Helsinki and genetic study protocols were approved by the institutional review boards of Kyoto Prefectural University of Medicine.

### Patients and T-ALL Subtypes

Thirty-one patients with confirmed T-ALL were diagnosed from January 1986 to September 2010 at the Kyoto Prefectural University of Medicine and affiliated hospitals. The diagnosis of T-ALL was based on the morphological features of bone marrow aspirates, and the immune-phenotypic analyses of leukemic cells expressing surface or cytoplasmic CD3. Of 31 T-ALL patients, 21 patients’ primay leukemic cells were stored. They were classified as ETP-ALL (n = 9) or typical T-ALL (n = 12). ETP-ALL was diagnosed using a previously described criteria [4]: lack of CD1a and CD8 expression, weak CD5 expression with less than 75%, and at least 25% expression of one or more myeloid or stem cell markers, including CD117, CD34, HLA-DR, CD13, CD33, CD11b and CD65. Molecular analyses and other biological studies were performed on RNA extracted from the primary leukemic cells.

### Real-Time Quantitative-PCR Analysis

Total RNA was extracted from the leukemic cells using the RNeasy Mini Kit (Qiagen, Venio, Netherlands) according to the manufacturer’s instructions. The cDNA for reverse transcriptase (RT)-PCR was synthesized using the SuperScript First-Strand Synthesis System (Invitrogen, Carlsbad, CA, USA) according to the manufacturer’s instructions. To compare the gene expression patterns of transcription factors related to the differentiation of myeloid and lymphoid cells in ETP-ALL with those in typical T-ALL, real-time quantitative-PCR (q-PCR) was performed on primary leukemic cells to determine the expression levels of the following transcription factors: *C/EBPα* and *ID2*, previously reported to be associated with myeloid differentiation [6, 7]; *NOTCH1*, *LYL1*, *IL7R* and *LMO2*, previously reported to be associated with leukemogenesis in T-ALL [8–10]; *MEF2C*, *PU*.*1*, and *FLT3*, previously reported to be associated with myeloid and lymphoid differentiation [11, 12].

q-PCR was conducted using the 7300 Real-Time PCR System (Applied Biosystems, Foster City, CA, USA) with SYBR Green 1 (Takara Bio, Tokyo, Japan). The relative target mRNA expression was determined using the comparative threshold (ΔC_T_) method. Glyceraldehyde-3-phosphate dehydrogenase (*GAPDH*) was used as an internal control. The primer pairs used in this study are listed in S1 Table.

### Cell Lines, Cell Culture, and Reagents

We used two human T-ALL cell lines (the *MEF2C*-expressing LOUCY and-non-expressing Jurkat cell lines), an interleukin 3 (IL-3) dependent murine pro-B cell line (BaF3), and WEHI-3 cells (myelomonocytic leukemia, Balb/C mouse cells). LOUCY cells were kindly provided by Dr. J. Meijerink (Sophia Children’s Hospital, Rotterdam, Netherlands) [13]. Jurkat cells and BaF3 cells were purchased from the American Type Culture Collection (Manassas, VA, USA). WEHI-3 cells were kindly provided by Dr. M. Suzuki (Tohoku University, Sendai, Japan) [14]. Plat-E packaging cells were kindly provided by Dr. T. Kitamura (The Institute of Medical Science, Tokyo University, Tokyo, Japan). Murine marrow stromal MS-5 cells were kindly provided by Dr. K. Ito (Kyoto University, Kyoto, Japan) [15].

The LOUCY, Jurkat, WEHI-3 and MS-5 cells were cultured in suspension in RPMI-1640 medium supplemented with 10% fetal bovine serum (FBS), penicillin (100 U/ml) and streptomycin (10 mg/ml), at 37°C, in a humidified atmosphere of 5% CO_2_. BaF3 cells were cultured in suspension in RPMI-1640 medium supplemented with 10% FBS, 20% of the supernatant in which the WEHI-3 cells were cultured, penicillin (100 U/ml), and streptomycin (10 mg/ml), at 37°C, in a humidified atmosphere of 5% CO_2_. PSL was purchased from Sigma-Aldrich (St. Louis, MO, USA), dissolved in DMSO, and stored as a 100 mM stock solution in small aliquots at -20°C. ABT-737 (a Bcl2 inhibitor) was purchased from Calbiochem (Billerica, MA, USA), dissolved in DMSO, and stored as a 100 μM stock solution in small aliquots at -80°C. PKC-412 (FLT3 inhibitor) was purchased from Sigma-Aldrich, dissolved in DMSO, and stored as 100 μM stock solutions in small aliquots at -80°C.

### Establishment of BaF3 Cell Lines Expressing MEF2C Protein

Retroviral constructs encoding *MEF2C* were generated by inserting the PCR fragment of *MEF2C* into the retroviral vector MSCVneo (Clontech, Mountain View, CA, USA). The fragment of *MEF2C* was sequenced in its entirety (Gene accession number: NM_002397.4). The production of retroviral supernatants in Plat-E cells was performed as previously described [16]. The infection of BaF3 cells was carried out as previously described [17], and the transduced BaF3 cells were cultured with G-418 (Takara Bio). The cells that survived after three passages were subjected to the experiments described below.

### Western Blotting

Cells were lysed with Laemmli sample buffer. Samples were boiled for 5 min in sample buffer containing bromophenol blue and 1×β-ME, and the proteins were separated by sodium dodecyl sulphate-polyacrylamide gel electrophoresis (SDS-PAGE). Electrophoretic separation was carried out on 15% polyacrylamide gels (Bio-Rad, Hercules, CA, USA), and proteins were subsequently transferred to an Immobilon-P PVDF transfer membrane (Millipore, Billerica, MA, USA). Membranes were blocked in PBS-Tween 20 (PBS-T) with 5% non-fat dry milk powder, and incubated with the primary antibodies β-actin (1:10000, Sigma-Aldrich) and MEF-2C (1:200, Santa Cruz Biotechnology, Santa Cruz, CA, USA). The membranes were then washed with PBS-T and incubated with anti-mouse or anti-goat secondary antibody (1:5000, Santa Cruz Biotechnology).

### Cell Proliferation Assay

Cell proliferation was measured with a WST cell viability and proliferation assay (Nacalai Tesque, Kyoto, Japan) according to the manufacturer’s instructions. The cells were seeded in a 96-well plate at 2×10^5^/well. Subsequently, the cells were cultured for 48 hours with serial concentrations of PSL and/or ABT-737 and/or PKC-412. Absorbance was measured after 48 hours by optical density absorption analysis at 450 nm using a multiplate reader (Multiskan JX, Thermo Fisher Scientific, Yokohama, Japan). The concentration of PSL and/or ABT-737 and/or PKC412 causing 50% growth inhibition (IC50) of leukemic cells was determined. The interaction of two compounds was quantified by determining the CI (combined index) according to the classic isobologram equation [18]. CI = (D)_1_/(Dx)_1_+(D)_2_/(Dx)_2_, where Dx is the dose of one compound alone required to produce an effect, and (D)_1_ and (D)_2_ are the dose of both compounds that produce the same effect. From this equation, the combined effects of two drugs can be assessed as either summative (additive or zero interaction) indicated as CI = 1, synergistic indicated as CI<1, or antagonistic indicated as CI >1.

### Apoptosis Assay

Apoptotic cell death was determined by Annexin V-FITC / propidium iodide (PI) staining using the Annexin V-FITC Apoptosis Detection Kit (R&D Systems, Minneapolis, MN, USA) according to the manufacturer’s instructions. Data were analyzed with Cell Quest software (BD Biosciences, Sparks, MD, USA).

### Co-Culture System with a Stromal Layer of MS-5 Cells

Murine marrow stromal MS-5 cells were plated in 6-well plates at 2.5×10^5^/well for 4 hours. Primary leukemic cells were added to the stromal cells 4 hours before PSL and/or ABT-737 was added to the medium. After treatment for 72 hours with PSL and/or ABT-737, the viability of primary leukemic blast cells was determined by Annexin V/PI staining (R&D Systems). Annexin (-)/PI (-) cells were defined as viable cells.

### Statistical Analysis

Statistical analysis was performed using the 2×2 chi-square test, Fisher’s test and Mann-Whitney U-test, as appropriate.

## Results

### The Gene Expression Pattern of Transcription Factors in ETP-ALL Cells

**Fig 1:**
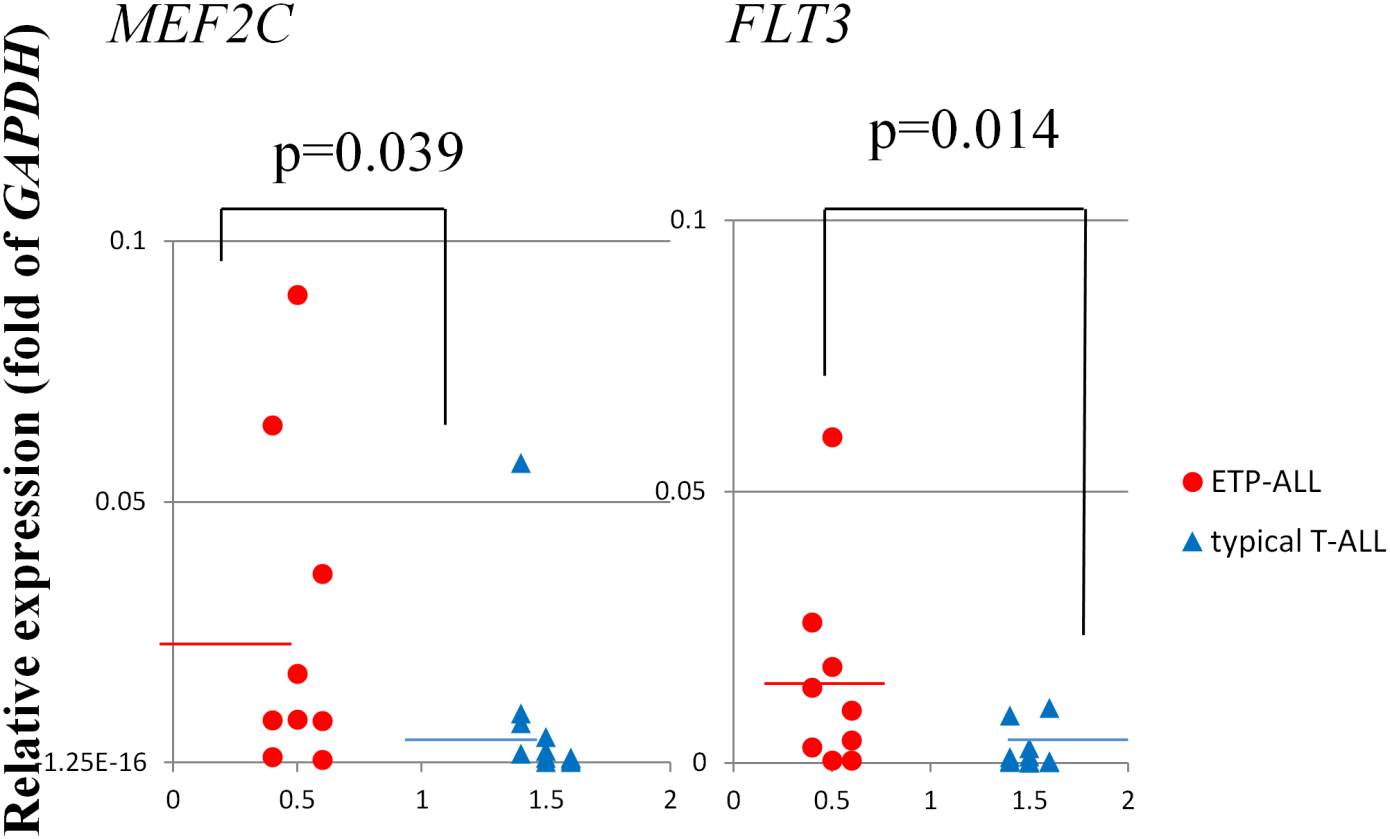
Expression levels of *MEF2C* and *FLT3* in ETP-ALL vs. typical T-ALL blast cells. Comparison of the expression levels of *MEF2C* and *FLT3* in ETP-ALL vs. typical T-ALL blast cells determined by real-time quantitative-PCR analysis.

ETP-ALL is considered to originate from the oncogenically transformed ETPs that are a subset of the thymocytes representing immigrants from the bone marrow with myeloid differentiation potential [4]. Thus we first evaluated the expression levels of *PU*.*1*, *C/EBPα* and *ID2*. However, there was no significantly higher expression of these transcription factors in the ETP-ALL cells. We also found no significant differences in the expression levels of *NOTCH1*, *LYL1*, *IL7R* and *LMO2* between the ETP-ALL and typical T-ALL cells (Table 1). q-PCR analysis demonstrated that *MEF2C* and *FLT3* were expressed at significantly higher levels in ETP-ALL than in typical T-ALL cells (*MEF2C*: p = 0.039, *FLT3*: p = 0.014) (Table 1, Fig 1). Although *FLT3* was overexpressed, no internal tandem duplications (ITD) of the juxtamembrane domain were detected (data not shown).

**Table 1:** The gene expression levels of transcription factors related to differentiation of lymphoid/ myeloid cells in ETP-ALL compared to typical T-ALL.

		ETP-ALL(n = 9)	typical T-ALL(n = 12)	P
*CEBPα*	median	0.000206	0.0000339	0.1217
	(range)	(0–0.00151)	(0–0.000383)	
*ID2*	median	0.0132	0.0139	0.5444
	(range)	(0.00622–0.0235)	(0.000307–0.0407)	
*NOTCH1*	median	0.0236	0.0273	0.4036
	(range)	(0.00680–0.0813)	(0.00383–0.0896)	
*LYL1*	median	0.000967	0.000228	0.1395
	(range)	(0–0.00449)	(0–0.00172)	
*IL7R*	median	0.0281	0.0192	0.513
	(range)	(0.00191–0.0890)	(0.000561–0.0854)	
*LMO2*	median	0.00365	0.00258	0.3332
	(range)	(0.000119–0.0156)	(5.35E-05–0.00936)	
*MEF2C*	median	0.0259	0.00724	0.039
	(range)	(0.000610–0.0896)	(9.51E-05–0.0575)	
*PU*.*1*	median	0.03	0.0204	0.2169
	(range)	(0.000610–0.0748)	(9.51E-05–0.117)	
*FLT3*	median	0.015	0.0021	0.0142
	(range)	(0.000378–0.0600)	(3.81E-05–0.0101)	

### BCL2 Inhibitor (ABT-737) Restored PSL Sensitivity in T-ALL Cell Lines with High Expression Levels of *MEF2C*

**Fig 2:**
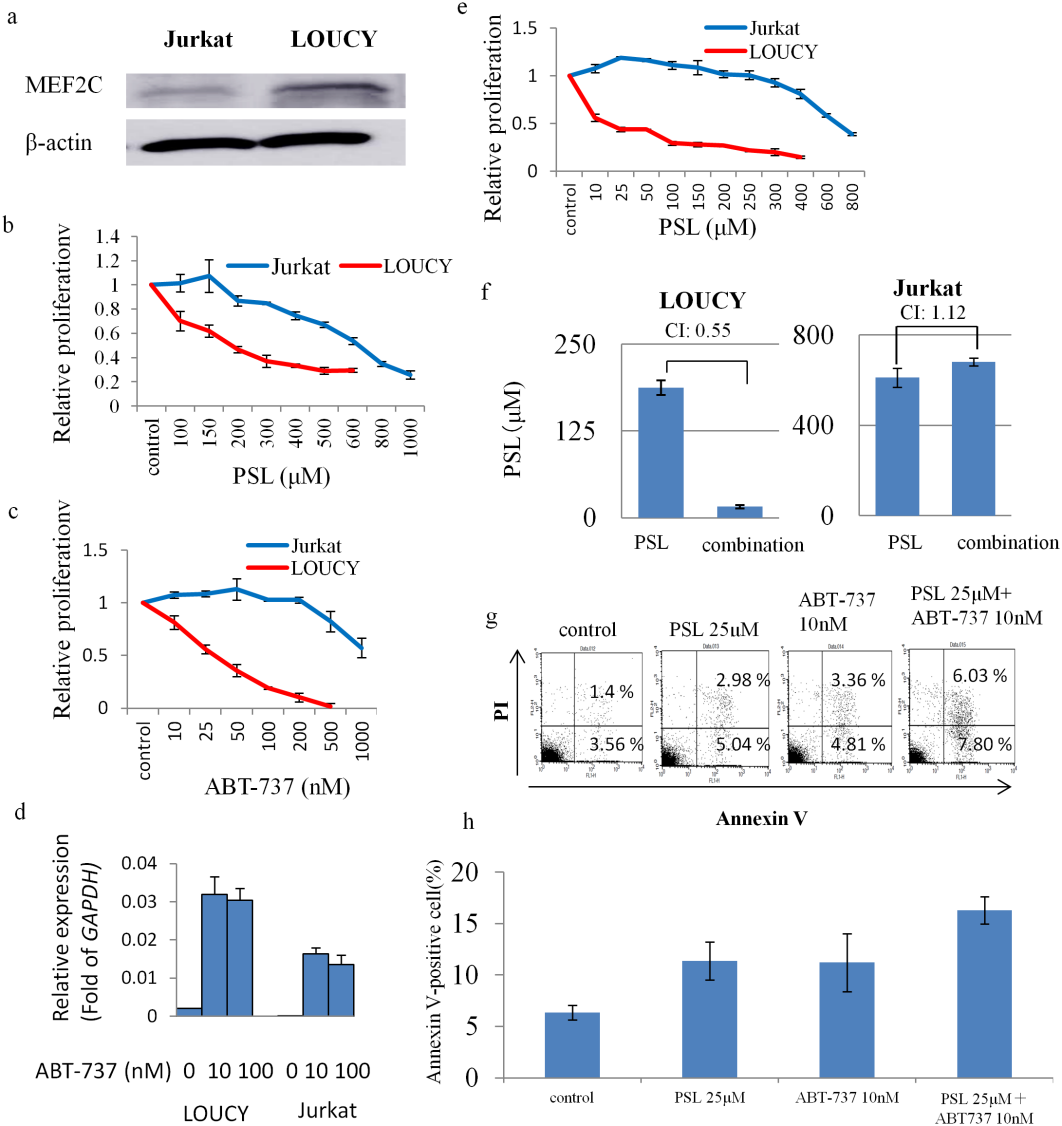
Comparison of LOUCY and Jurkat leukemia cells. (a) Expression of MEF2C and β-actin by western blot analyses. (b), (c) Cell growth inhibition of leukemia cells assessed by WST assay with serial concentrations of prednisolone (PSL) or ABT-737. (d) q-PCR analysis of BCL2 of two cell lines 12 hours after treatment of ABT-737 with the concentration of 10 or 100 nM. The bar indicates the mean±SE of two independent experiments in triplicate (e) Cell growth inhibition with serial concentrations of prednisolone (PSL) combined with ABT-737 (10 nM). (f) Calculation of the IC50 of PSL, following treatment of leukemic cells with either PSL, or a combination of PSL and ABT-737. Calculated combination Index of less than 1.0 was considered as a synergistic effect. (g), (h) Apoptosis study. Annexin V-positive cells were counted as apoptotic. Flow cytometry data in (g) and the mean±SE of three independent experiments in (h).

We tested if high expression levels of *MEF2C* were directly associated with PSL resistance in T-ALL cells because MEF2C may augment BCL2 activity to inhibit apoptosis [19], and be responsible for the poor responsiveness to the initial treatment of T-ALL with PSL. Thus we comparatively evaluated the sensitivity of two human T-ALL cell lines, LOUCY and Jurkat (*MEF2C* expression was positive in LOUCY, but not in Jurkat) to PSL (Fig 2a). Our study revealed that both cell lines were intrinsically resistant to PSL with an IC50 (μM) of LOUCY 188±18.3 vs. Jurkat 612±42.0 (Fig 2b), suggesting that the high expression of *MEF2C* was not the only mechanism for PSL resistance. Next, we tested if the pharmacological inhibition of BCL2 activity resulted in the restoration of PSL sensitivity in the T-ALL cells with high *MEF2C* expression. LOUCY cells became sensitive to ABT-737 when the agent was added to the culture medium (IC50 of ABT-737: 32.8±10.9 nM, Fig 2c). q-PCR analysis revealed that basal *BCL2* expression level was much higher than that in Jurkat (Fig 2d). This finding was consistent with the previous report [20]. When the cells were treated with ABT-737, the expression levels of *BCL2* were increased in both cell lines (LOUCY > Jurkat), suggesting that inhibitory effect of ABT-737 in protein level induced increase of mRNA expression of *BCL2* (Fig 2d). In addition, the combined treatment of PSL with ABT-737 resulted in a significant reduction in the IC50 of PSL in LOUCY cells, with a CI value of 0.55 (Fig 2e and 2f). By contrast, Jurkat cells remained resistant to ABT-737 (IC50 of ABT-737: 955±9.3 nM, Fig 2c), as well as to a combination treatment, with a CI value of 1.12 (Fig 2e and 2f). LOUCY cells treated with PSL (25 μM) and/or ABT-737 (10 nM) were examined for apoptosis with the Annexin V/PI assay, and the number of Annexin V positive cells was higher in the cells treated with both agents (Fig 2g and 2h). These findings suggest that LOUCY cells restore PSL sensitivity with a pharmacological dose of the BCL2 inhibitor, ABT-737.

### BCL2 Inhibitor (ABT-737) Sensitizes *MEF2C-Transduced* BaF3 Cells to PSL

**Fig 3:**
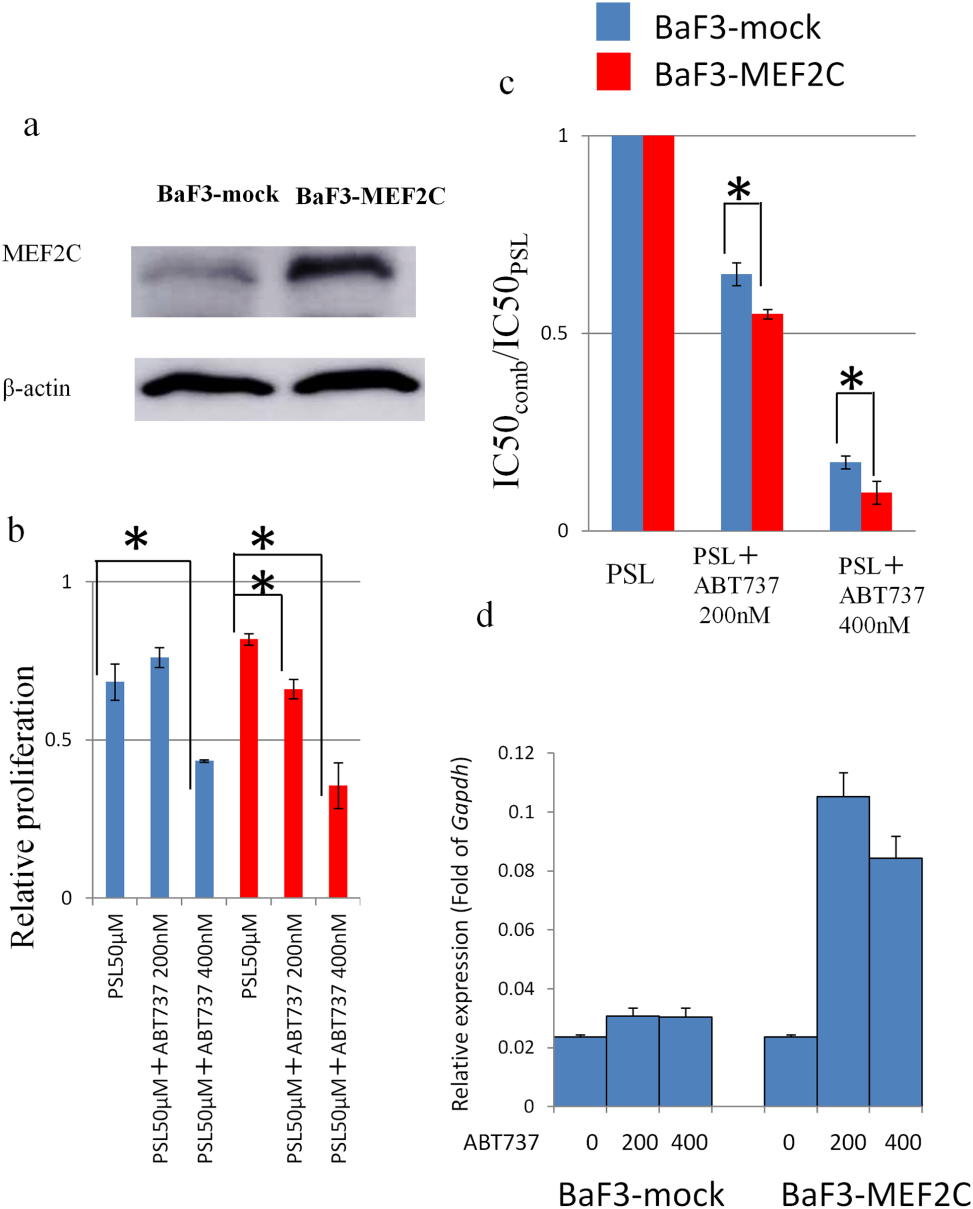
Comparison of BaF3-MEF2C vs. BaF3-mock cells. (a) Expression of MEF2C and β-actin determined by western blot analysis. (b) The number of viable cells assessed using WST assay following incubation, with PSL (50 μM) and/or ABT-737 (200 and 400 nM). (c) Calculation of the IC50 of PSL when treated with PSL alone, or with a combination of PSL and ABT-737 (200 or 400 nM). P-value of less than 0.05 was considered statistically significant. *: p< 0.05. (d) q-PCR analysis of Bcl2 of two cell lines 12 hours after treatment of ABT-737 with the concentration of 200 or 400 nM. The bar indicates the mean±SE of two independent experiments in triplicate.

To assess the correlation of a high expression of *MEF2C* with PSL resistance, and the role of BCL2 inhibition in the reversal of PSL resistance, we generated BaF3 cells with a stable expression of *MEF2C* (BaF3-MEF2C). Western blot analysis confirmed sufficient expression of MEF2C in Plat-E packaging cells transfected with MSCV-MEF2C (Fig 3a). BaF3-MEF2C was less sensitive to PSL, but not significantly (Fig 3b). Treatment with PSL (50 μM) and/or ABT-737 (200 nM or 400 nM) inhibited the proliferation of BaF3-MEF2C cells more than that observed in the BaF3-mock cells, with a significant reduction in the IC50 values (Fig 3b and 3c). q-PCR analysis revealed the basal expression level of *Bcl2* in BaF3 ± MEF2C was not statistically different. However, when the cells were treated with ABT-737, induction of *Bcl2* expression was more prominent in BaF3 expressing MEF2C. These findings might explain that exogenous expression of MEF2C sensitized BaF3 cells to ABT-737 (Fig 3d).

### BCL2 Inhibitor (ABT-737) Restores PSL Sensitivity in Primary Leukemic Blast Cells of ETP-ALL Patients

**Fig 4:**
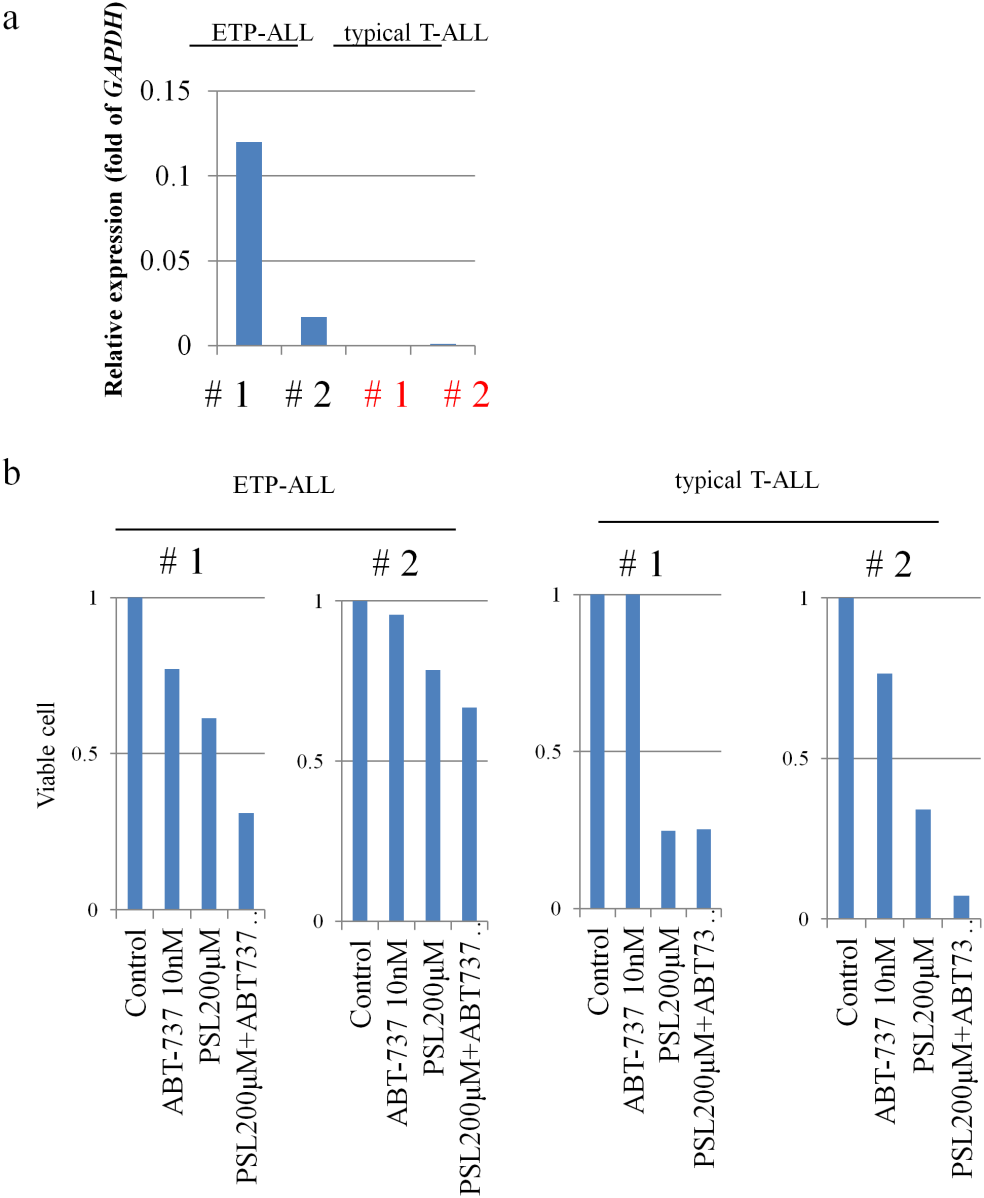
*MEF2C* expression and PSL sensitivity of primary leukemic blast cells in four T-ALL patients (two ETP-ALL and two non-ETP ALL). (a) *MEF2C* expression levels determined by real-time quantitative-PCR. *GAPDH* was used as an internal control. (b) Viability of cells was counted after treatment with PSL (200 μM) and/or ABT-737 (10 nM). Annexin (-)/PI(-) cells were defined as viable cells.

Based on the BaF3 cell data, we also tested the effect of PSL and/or ABT-737 on the primary leukemic blast cells obtained from four T-ALL (two EPT-ALL and two typical T-ALL) patients. Primary leukemic cells from typical T-ALL patients were confirmed to show low levels of expression of *MEF2C*, while those from ETP-ALL patients showed high levels of expression of *MEF2C* (Fig 4a). Treatment with PSL (200 μM) caused more than a 50% reduction in the number of viable primary leukemic blast cells in the typical T-ALL blast cells, but not in the ETP-ALL blast cells (Fig 4b), which is consistent with the PSL response shown in their clinical course (data not shown). ABT-737 (10 nM) alone did not induce more than a 50% reduction in the number of primary leukemic blast cells of EPT-ALL patients. However, a combination treatment of PSL (200 μM) and ABT-737(10 nM) reduced the viability to <50% of the primary leukemic blast cells in one ETP-ALL patient (Fig 4b).

### FLT3 Inhibitor (PKC-412) Did Not Restore PSL Sensitivity in T-ALL Cell Lines with High Expression Levels of *MEF2C*

**Fig 5:**
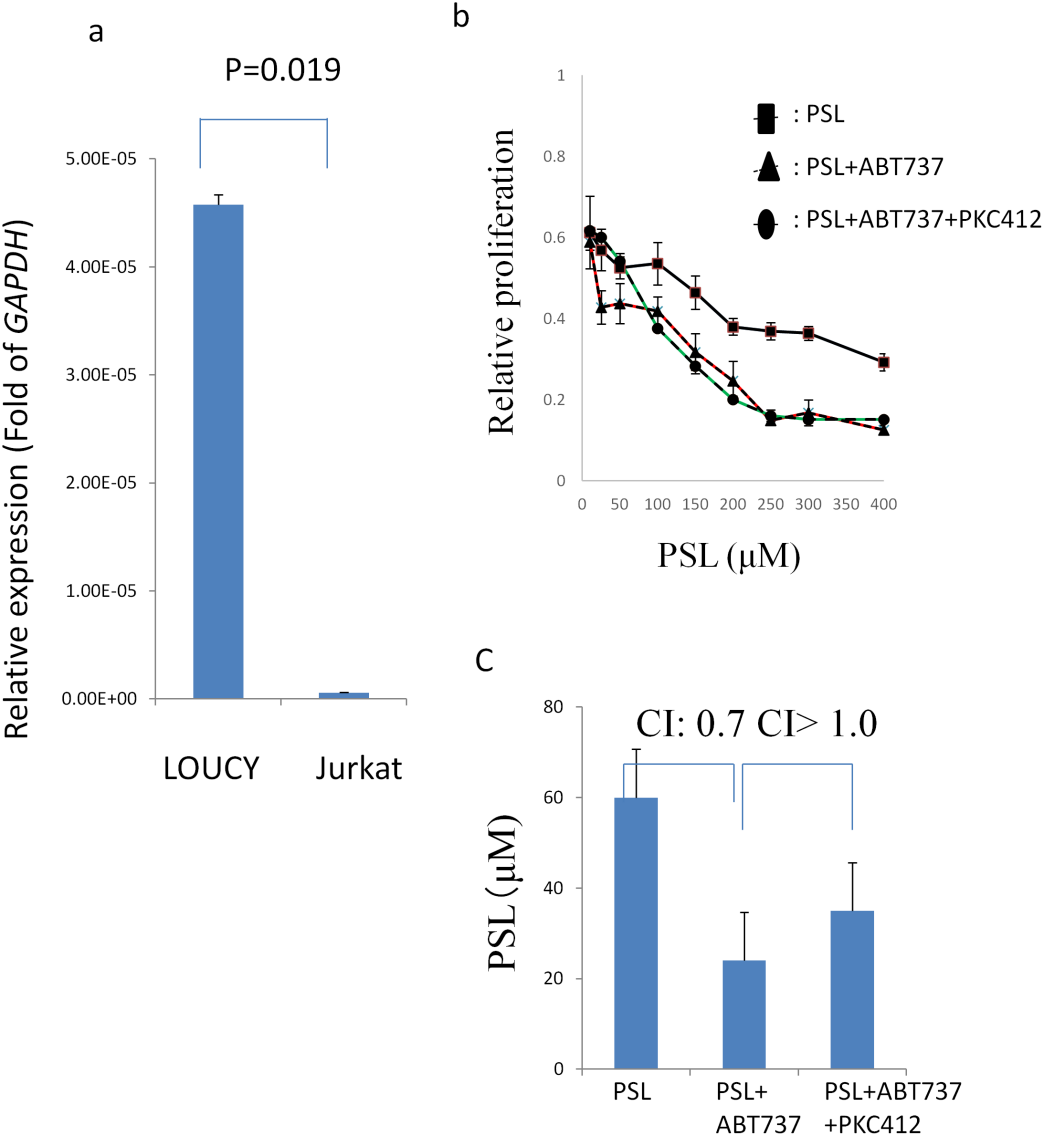
*FLT3* expression and PSL sensitivity of LOUCY cells treated with ABT-737 in combination with PKC-412. (a) *FLT3* expression levels determined by real-time quantitative-PCR. *GAPDH* was used as an internal control. (b) Cell growth inhibition with serial concentrations of prednisolone (PSL) combined with ABT-737 (10 nM) and/or PKC-412 (100 nM). (c) Calculation of the IC50 of PSL, following treatment of leukemic cells with PSL, a combination of PSL and ABT-737, and a combnation of PSL, ABT-737 and PKC412.

Finally, we investigated whether FLT3 inhibitor (PKC412) in combination with ABT737 treatment restore PSL sensitivity more prominently in T-ALL cells with high expression of *MEF2C*. First, we determined *FLT3* expression was much higher in LOUCY than in Jurkat by q-PCR analysis (Fig 5a). However, the expression level of *Flt3* was not different in BaF3 irrespective of MEF2C expression (data not shown), suggesting *Flt3* expression might be silenced in BaF3 cells. Thus, the effect of PKC-412 was investigated in LOUCY and Jurkat. In line with the expression level of *FLT3*, LOUCY was more sensitive to PKC-412 than Jurkat (IC50: 667 vs 1807 nM). Then, we tested if the combination of PSL and PKC412 resulted in the restoration of PSL sensitivity in LOUCY cells. However, PKC412 antagonized PSL in LOUCY cells (IC50 of PSL: 542 vs 60μM, CI>1.0). Finally, we tested if the combination of PKC412 and ABT737 resulted in the restoration of PSL sensitivity in LOUCYcells. The combined treatment of PKC-412 with ABT-737 did not result in a significant reduction in the IC50 of PSL in LOUCY cells (IC50 of PSL: 35 vs 24μM) (Fig 5b and 5c).

## Discussion

ETP-ALL is considered to be a neoplasm of less mature hematopoietic progenitor or stem cells that arrest at a very early maturational stage and retain the capacity for myeloid differentiation [21]. At the molecular level, high expression levels of *MEF2C* and *FLT3* may characterize ETP-ALL. *MEF2C* is a member of the MADS-box transcription factor family that includes the *MEF2A-D* genes, which are important regulators of skeletal muscle development [22]. In the hematopoietic system, *MEF2C* is abundantly expressed in both hematopoietic stem cells and common myeloid progenitors or common lymphoid precursors [23]. *MEF2C* is expressed in normal human thymocyte pre-DN1 and DN1 subsets, but its expression is dramatically decreased beyond the DN2 stage [13]. These findings suggest that *MEF2C* is a central regulator for normal early T-cell development. Homminga *et al*. first revealed that the expression level of *MEF2C* was high in immature T-ALL cases, as defined by gene expression profiling [13]. More recently, they determined that these ALL patients belong to an ETP-ALL entity, with respect to their biology and genetics [24]. Neumann *et al*. also reported that expression of *MEF2C* was significantly higher in ETP-ALL compared to typical T-ALL in adults [25]. As direct target genes for *MEF2C*, *LYL1* and *LMO2* were identified [13]. However, high or variable expression of *LYL1* or *LMO2* was described in ETP-ALL [4, 24]. On the other hand, *FLT3* is one of the most frequently mutated genes in acute myeloid leukemia. *FLT3* was also reported be overexpressed in ETP-ALL in adults [25]. *FLT3* mutations occur either by ITD of the juxtamembrane domain, or by point mutations that usually involve the kinase domain.

We found that *MEF2C* and *FLT3* were both expressed at higher levels in ETP-ALL than in typical T-ALL patients (*MEF2C*: p = 0.039, *FLT3*: p = 0.014) (Table 1, Fig 1). Also, in our cohort, we found that the expression levels of *LYL1* or *LMO2* were not significantly higher in ETP-ALL compared to those of typical T-ALL patients. Although previous reports show a high rate of *FLT3* mutations including FLT3-ITD and D835 (35%) in EPT-ALL [21, 26], we detected no FLT3-ITD in our patients (data not shown).

Taken together, our findings further confirm that ectopic expression of *MEF2C* or *FLT3* in ETPs leads to a differentiation arrest, and results in the development of ETP-ALL. Clinically, as well as in the *in vitro* system, ETP-ALL cells were resistant to PSL, in correlation with a poor clinical outcome of this distinct subset of T-ALL [4]. We showed in this study that the BCL2 inhibitor (ABT-737) restored the PSL sensitivity in primary leukemic cells with a high expression of *MEF2C*, as well as in *MEF2C-*transduced BaF3 cells. In addition, we demonstrated that treatment of ABT-737, in combination with PSL, accelerated the killing of primary leukemic blast cells of ETP-ALL more profoundly than the treatment of PSL alone. These findings suggest that BCL2 inhibitor restores *in vitro* PSL sensitivity in ETP-ALL cells. There was a report that BCL2-regulated apoptosis by *MEF2C* is repressed by Nur77, which induces apoptosis by the conversion of BCL2 from anti-apoptotic to pro-apoptotic [26]. Similarly, we hypothesize that the BCL2 inhibitor may block the anti-apoptotic effect of *MEF2C*, and thus restore the sensitivity to PSL in ETP-ALL cells. Recently, the pharmacological inhibition of the BCL2 family of proteins emerges as a promising therapeutic measure for hematological malignancies [27–30]. Based on these data, inhibition of BCL2 might become an *in vivo* therapeutic candidate for ETP-ALL patients. Preclinical studies are required for confirming the effectiveness of BCL2 inhibition in the treatment of ETP-ALL.

Finally, FLT3 inhibitor might be also promising agent for ETP-ALL [26]. We determined PKC-412 was more effective in T-ALL cell line with FLT3 high expression. However, we determined PKC412 antagonized growth inhibition of PSL in LOUCY cells even when used in combination with PSL and ABT737. Although precise mechanism of this antagonized effect of PKC412 on PSL sensitivity was not determined, we should be cautious to decide which type of molecular targeting agents to introduce in conventional treatment regimens.

## Supporting Information

S1 Table PCR primers used in this study. C/EBPα: CCAAT/enhancer-binding protein alpha. GAPDH: glyceraldehyde-3-phosphate dehydrogenase.
(DOCX)
