# Supplementary material for: BCL2 Inhibitor (ABT-737): A Restorer of Prednisolone Sensitivity in Early T-Cell Precursor-Acute Lymphoblastic Leukemia with High MEF2C Expression?
Source: PLoS One. 2015 Jul 14;10(7):e0132926. doi: 10.1371/journal.pone.0132926 (PMC4501565; doi:10.1371/journal.pone.0132926)
Supplement: S1 Table — C/EBPα: CCAAT/enhancer-binding protein alpha. GAPDH: glyceraldehyde-3-phosphate dehydrogenase. (DOCX) [file pone.0132926.s001.docx]

| **Supplementary Table 1** PCR primers used in this study | | |
| --- | --- | --- |
|  | Direction | Primer |
| C/EBPα | Forward | 5'-TTCAACGACGAGTTCCTG |
|  | Reverse | 5'-GCCCGGGTAGTCAAAGTC |
| ID2 | Forward | 5’-ACGGATATCAGCATCCTGTC |
|  | Reverse | 5’-CCGTGAATTTGTTGTTGTTG |
| NOTCH1 | Forward | 5’-GAGGTCAATGAGTGCAACAG |
|  | Reverse | 5’-GACACAAGGGTTGGATTCAC |
| LYL1 | Forward | 5’-ACTCTCCACCCTGGGAACT |
|  | Reverse | 5’-CTCACAGTGGCTTGGTCTC |
| IL7R | Forward | 5’-TCATCTTGGCCTGTGTGTTA |
|  | Reverse | 5’-CACCCTATGAATCTGGCAGT |
| LMO2 | Forward | 5’-TGAAGGCCATCGACCAGTA |
|  | Reverse | 5’-CTTTGTCTTTCACCCGCATT |
| MEF2C | Forward | 5’-CCCCCAATGAATTTAGGAAT |
|  | Reverse | 5’-AGTTGCTACGGAAACCACTG |
| PU.1 | Forward | 5'-GAAGACCTGGTGCCCTATGA |
|  | Reverse | 5'-CTCTGGAGCTCCGTGAAGTT |
| FTL3 | Forward | 5’-CATGCAGAAAATGATGATGC |
|  | Reverse | 5’-CTTCCAGGTCCAAGATGGTA |
| BCL2 | Forward | 5’-ATGTGTGTGGAGAGCGTCAACC |
|  | Reverse | 5’-AGCCAGGAGAAATCAAACAGAGG |
| Flt3 | Forward | 5’-GAAGACCTCCTTTGCTTTGCG |
|  | Reverse | 5’-TGACCAACACATTCCTGGCTGC |
| GAPDH | Forward | 5'-GCACCGTCAAGGCTGAGAAC |
|  | Reverse | 5'-ATGGTGGTGAAGACGCCAGT |
| Gapdh | Forward | 5’- GATGGGTGTGAACCACGAGA |
|  | Reverse | 5’- AGTGATGGCATGGACTGTGG |
| C/EBPα: CCAAT/enhancer-binding protein alpha. | | |
| GAPDH: glyceraldehyde-3-phosphate dehydrogenase. | | |
